# Scalable production of homogeneous cardiac organoids derived from human pluripotent stem cells

**DOI:** 10.1016/j.crmeth.2023.100666

**Published:** 2023-12-18

**Authors:** Taijun Moriwaki, Hidenori Tani, Kotaro Haga, Yuika Morita-Umei, Yusuke Soma, Tomohiko C. Umei, Otoya Sekine, Kaworu Takatsuna, Yoshikazu Kishino, Hideaki Kanazawa, Jun Fujita, Keiichi Fukuda, Shugo Tohyama, Masaki Ieda

**Affiliations:** 1 Department of Cardiology, Keio University School of Medicine, Shinjuku, Tokyo, Japan; 2 Joint Research Laboratory for Medical Innovation in Heart Disease, Keio University School of Medicine, Shinjuku, Tokyo, Japan; 3 Kanagawa Institute of Industrial Science and Technology (KISTEC), Kawasaki, Kanagawa, Japan; 4 Department of Pathology & Immunology, Baylor College of Medicine, Houston, TX, USA

**Keywords:** human induced pluripotent stem cells, cardiac spheroids, cardiac organoids, drug discovery, regenerative therapy, large-scale culture

## Abstract

Three-dimensional (3D) cultures are known to more closely mimic *in vivo* conditions compared with 2D cultures. Cardiac spheroids (CSs) and organoids (COs) are useful for 3D tissue engineering and are advantageous for their simplicity and mass production for regenerative therapy and drug discovery. Herein, we describe a large-scale method for producing homogeneous human induced pluripotent stem cell (hiPSC)-derived CSs (hiPSC-CSs) and COs without scaffolds using a porous 3D microwell substratum with a suction system. Our method has many advantages, such as increased efficiency and improved functionality, homogeneity, and sphericity of hiPSC-CSs. Moreover, we have developed a substratum on a clinically relevant large scale for regenerative therapy and have succeeded in producing approximately 40,000 hiPSC-CSs with high sphericity at once. Furthermore, we efficiently produced a fused CO model consisting of hiPSC-derived atrial and ventricular cardiomyocytes localized on opposite sides of one organoid. This method will facilitate progress toward hiPSC-based clinical applications.

## Highlights

• We develop a scalable method to produce hiPSC-CSs/COs
• The method employs suction to produce functionally homogeneous hiPSC-CSs
• Use of a large substratum yields ∼40,000 homogeneous hiPSC-CSs
• The method fabricates hiPSC-COs with localized atrial and ventricular tissue

## Motivation

We previously developed systems for producing cardiac spheroids (CSs) from metabolically purified human induced pluripotent stem cell (hiPSC)-derived cardiomyocytes (hiPSC-CMs) for regenerative therapy. For safety and efficacy, it is imperative to avoid the occurrence of internal necrosis within hiPSC-CSs for transplantation. Furthermore, for drug discovery, there is a need to generate hiPSC-derived CSs and cardiac organoids (hiPSC-CSs/COs) with low variability in terms of drug responsiveness. To address these challenges, we developed the “suction method” by leveraging the ability of the porous substratum to produce a large number of highly homogeneous and functional hiPSC-CSs/COs. The developed method will facilitate the production of large amounts of hiPSC-CSs/COs for regenerative therapy and drug discovery.

## Graphical abstract

**Figure:**
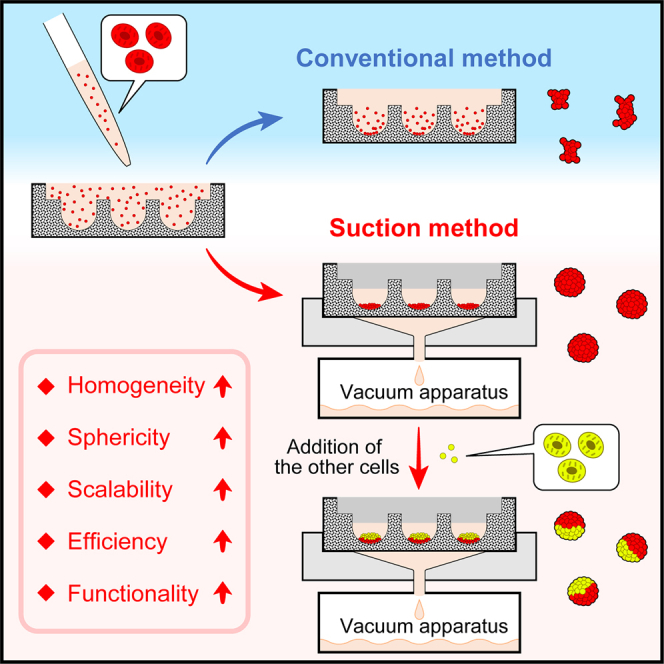

## Teaser

Moriwaki et al. developed a “suction method” by taking advantage of the characteristics of the porous substratum to produce a large number of highly homogeneous and functional human induced pluripotent stem cell-derived cardiac spheroids (hiPSC-CSs) and cardiac organoids (COs) for hiPSC-based drug discovery and regenerative therapy.

## Introduction

Three-dimensional (3D) cell cultures are known to more closely mimic *in vivo* conditions compared to 2D cell cultures because of the robust interactions fostered between cells and the extracellular matrix.^1^^,^^2^ Therefore, they are more applicable for disease modeling, assessing drug efficacy, and toxicity testing. In the field of cardiology, 3D tissue engineering techniques can induce the maturation of human induced pluripotent stem cell (hiPSC)-derived cardiomyocytes (CMs), overcoming one of the main challenges of hiPSC-CMs, which is their immaturity.^3^^,^^4^^,^^5^ Notably, scaffold-free 3D models such as cardiac spheroids (CSs) and cardiac organoids (COs) self-assemble, are easy to fabricate, and do not require specific equipment. As an established disease model, COs with hypoxia and chronic adrenergic stimulation have been reported to recapitulate the structure and function of myocardial ischemia.^6^ Moreover, CSs/COs are useful for drug toxicity tests. CSs/COs also offer the advantage of being amenable to mass production.

We have developed systems for producing CSs from metabolically purified hiPSC-derived ventricular CMs and transplanting them into the heart to treat patients with refractory severe heart failure.^7^^,^^8^^,^^9^^,^^10^^,^^11^^,^^12^^,^^13^ Although the retention rate of single-cell CMs transplanted into the heart is very low, we have succeeded in significantly improving the retention rate by producing hiPSC-derived CSs (hiPSC-CSs) of approximately 150 μm in size and transplanting them into the heart.^7^^,^^8^^,^^14^^,^^15^ In general, simplicity and mass production are important for hiPSC-CS transplantation. Spheroid transplantation experiments have been conducted in other tissues such as the liver, bone, cartilage, spine, and brain to promote tissue regeneration and angiogenesis.^16^^,^^17^^,^^18^^,^^19^^,^^20^^,^^21^^,^^22^^,^^23^^,^^24^^,^^25^^,^^26^ It has been reported that spheroid formation enhances cell-specific functions in tissues and is now used in a wide range of applications, such as drug discovery and research on pathological mechanisms.^1^^,^^27^^,^^28^^,^^29^^,^^30^ There is also potential value in producing highly homogeneous and functional spheroids to evaluate the response to drugs more reproducibly.

Many researchers have developed organoid production methods.^31^^,^^32^^,^^33^^,^^34^^,^^35^^,^^36^ There are two major methods for organoid production: the first is to produce one spheroid per one well, and the second is to produce multiple spheroids per one well or vessel. One of the representative methods in the former group is to produce organoids using 96-well plates or 384-well plates with non-adhesive treatment.^32^ Although this method has the disadvantage that a large number of organoids cannot be produced, highly homogeneous organoids can be efficiently produced via centrifugation after cell seeding. One notable technique within the latter category involves the utilization of bioreactors and non-adhesive-surface-treated microwells.^8^^,^^24^^,^^37^^,^^38^^,^^39^^,^^40^^,^^41^ Bioreactors offer a practical approach for generating a substantial quantity of spheroids, characterized by a relatively uniform structure. These spheroids can be effectively guided to undergo differentiation into CMs originating from hiPSC spheroids.^39^ Microwell plates can be combined with centrifugation to produce spheroids with enhanced uniformity; however, given the necessity of centrifugation, it is not possible to produce large quantities of spheroids simultaneously. Therefore, we aim to develop an alternative technique that can yield more homogeneous spheroids without reliance on centrifugation while also facilitating the simultaneous production of a large number of spheroids. In light of these considerations, we adopted the latter method, which enables the production of multiple spheroid/organoids in a single culture system, ensuring scalability and simplicity. Moreover, we developed a method that satisfies the abovementioned requirements and can produce homogeneous spheroids.

In this study, we developed a “suction method” by taking advantage of the characteristics of the porous substratum to produce a large number of highly homogeneous and functional hiPSC-CSs for hiPSC-based drug discovery and regenerative therapy. Moreover, we developed a clinically relevant, larger substratum that can produce approximately 40,000 hiPSC-CSs for regenerative therapy. We also produced hiPSC-COs consisting of various types of cells by using this suction method.

## Results

### Suction method parameters for production of homogeneous hiPSC spheroids

**Figure 1:**
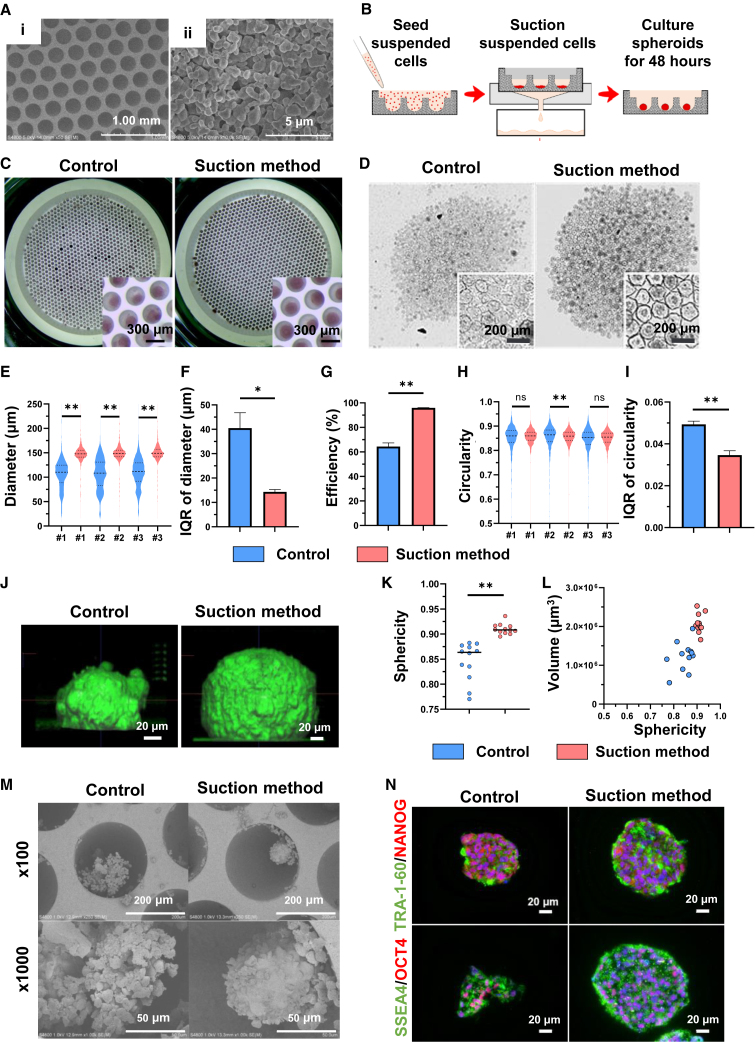
Suction method efficiently produces homogeneous hiPSC spheroids with high sphericity (A) Scanning electron microscope (SEM) images of the substratum. (i) Numerous microwells are present in the substratum. (ii) Microstructure of the substratum. (B) Schematic of the suction method. (C) Spheroids on substratum as revealed by alkaline phosphatase stain. (D) Bright-field microscopy images of hiPSC spheroids by the control method and the suction method. (E) Violin plot of the diameter of hiPSC spheroids. #1–3 indicate the experiment number. Brunner-Munzel test, #1: n = 1,065 spheroids, #2: n = 1,096 spheroids, and #3: n = 1,118 spheroids. (F) Interquartile range (IQR) of the diameter of iPSC spheroids. Welch’s test, n = 3. (G) Efficiency of hiPSC spheroid production, defined as the percentage of spheroids with diameters larger than 100 μm. Welch’s t test, n = 3. (H) Violin plot of the circularity of hiPSC spheroids. #1–3 indicate the experiment number. Brunner-Munzel test, #1: n = 1,065 spheroids, #2: n = 1,096 spheroids, and #3: n = 1,118 spheroids. (I) IQR of hiPSC spheroid circularity. Welch’s t test, n = 3. (J) 3D image of hiPSC spheroids observed using 3D imaging with Cell^3^iMager Estier. (K) The sphericity of hiPSC spheroids. Welch’s t test, n = 12 spheroids. (L) The sphericity and volume of hiPSC spheroids were presented on a scatterplot (n = 12 spheroids). The IQR of sphericity for the control method was 0.0571, whereas for the suction method, it was 0.0135. Similarly, the IQRs of volume were 4.25 × 10^5^ μm^3^ for the control method and 2.89 × 10^5^ μm^3^ for the suction method. (M) Representative SEM images showing hiPSC spheroids on the substratum. (N) Representative immunofluorescence images for TRA-1-60 (green), NANOG (red), SSEA4 (green), and OCT4 (red). Nuclei were stained with Hoechst 33342. The hiPSC data were evaluated using the 253G4 cell line. Data are presented as the mean ± SD. ∗p < 0.05; ∗∗p < 0.01.

There are two important factors for the preparation of homogeneous spheroids in multi-well culture systems. First, the same number of cells should be in each well; that is, cells should settle in each well within a short period. Second, the cells in the wells must be aggregated together in one place. We assumed that ideal spheroids would be produced by meeting these two requirements. We focused on a porous substratum made of ceramics.^42^^,^^43^ Ceramics are mainly used for artificial joints, tooth roots, and bones and are regarded as materials with high biocompatibility.^44^^,^^45^^,^^46^ Importantly, this substratum has numerous microscopic holes of approximately 200 nm in diameter that allow only the medium to pass through the substrate without loss of cells. The pore structure of each well was observed by scanning electron microscopy (SEM) (Figure 1A). We assumed that this property of the substratum would enable efficient production of homogeneous spheroids by the application of negative pressure downward to the cell suspension on the substratum with a vacuum device, causing the cells to aggregate at the bottom of the substratum. One spheroid was observed in each microwell 24–72 h after starting the culture of cells with 1 min suction (Figure 1B**)**. We called this method the “suction method” and evaluated whether it was possible to use this method to efficiently produce homogeneous spheroids in comparison with the conventional spontaneous sedimentation method as a control method. We then investigated the optimal degree of suction force because it can affect the morphology and homogeneity of hiPSC spheroids. We observed the spheroids under suction forces of −0.025, −0.050, and −0.075 MPa. The median diameter of the hiPSC spheroids was the same under all conditions; however, the deviation in diameter was significantly larger under −0.075 MPa than under −0.025 and −0.050 MPa, which was visible in the observation images (Figures S1A–S1C). There was no significant difference in the diameter deviation between hiPSC spheroids prepared at −0.025 and −0.050 MPa (Figure S1C). The efficiency of hiPSC spheroid production exhibited a notably higher performance when subjected to a pressure of −0.075 MPa compared to conditions of −0.025 and −0.050 MPa. However, no statistically significant distinction was observed between hiPSC spheroids prepared at pressures of −0.025 and −0.050 MPa (Figure S1D). The circularity of hiPSC spheroids remained consistent across all conditions, and the interquartile range (IQR) of circularity exhibited no significant difference between the all conditions (Figures S1E and S1F). Furthermore, the application of a pressure of −0.050 MPa facilitated the aspiration of the cell suspension onto the substratum in less than 1 min, while the process took approximately 3 min under a pressure of −0.025 MPa. These findings led us to proceed with further experiments employing a suction force of −0.050 MPa.

### Suction method efficiently produces homogeneous hiPSC spheroids with high sphericity

We next evaluated the morphological homogeneity of hiPSC spheroids produced by the conventional spontaneous sedimentation method (control method) and the suction method using 253G4 hiPSC lines. Since the sequential investigation demonstrated that hiPSC spheroids were formed stably with little deviation 2 days from the start of culture, we investigated the homogeneity at day 2 (Figures S1G–S1K). hiPSC spheroids were successfully formed on day 2, and the suction method yielded hiPSC spheroids with significantly larger diameters than the control method (Figures 1C–1E). The degree of diameter deviation was significantly smaller in the suction method than in the control method (Figure 1F). The efficiency of spheroid production was significantly higher when using the suction method than when using the control method (Figure 1G). While the circularity of hiPSC spheroids generally demonstrated a tendency to be higher in the suction method compared to the control method, there were variations among individual spheroids, with some displaying noteworthy differences while others did not (Figure 1H). The deviation in circularity was significantly smaller in the suction method than in the control method (Figure 1I). We also noninvasively evaluated the steric structure of hiPSC spheroids with 3D imaging using a Cell^3^iMager Estier (Screen Holdings, Kyoto, Japan) (Figure 1J). The sphericity of hiPSC spheroids was significantly higher in the suction method (Figure 1K). Upon analyzing scatterplots depicting the sphericity and volume of the hiPSC spheroids, it became evident that the deviation in both sphericity and volume was reduced when utilizing the suction method (IQRs of sphericity: 0.0571 in the control method and 0.0135 in the suction method, IQRs of volume: 4.25 × 10^5^ μm^3^ in the control method and 2.89 × 10^5^ μm^3^ in the suction method) compared to the control method (Figure 1L). These findings suggest that hiPSC spheroids produced via the suction method exhibit higher homogeneity and closer adherence to a spherical shape compared to those produced by the control method. Detailed observation of hiPSC spheroids on the substratum by SEM showed that hiPSC spheroids obtained by the suction method had a dense cell structure and were located with one per well, while those obtained by the control method had a sparse cell density and were located in multiples with a deviation in size (Figure 1M). This suggests that the higher aggregation of cells by the suction method may contribute to reducing morphological variability. We immunostained hiPSC spheroids with undifferentiated stem cell markers TRA-1-60, NANOG, SSEA4, and OCT4 to evaluate whether they maintained their undifferentiated status and found no obvious differences between the two methods, revealing that suction did not affect maintenance of the undifferentiated status (Figure 1N). These results suggest that the suction method can efficiently produce homogeneous hiPSC spheroids with high sphericity.

### Suction method efficiently produces homogeneous hiPSC-CSs with high sphericity

**Figure 2:**
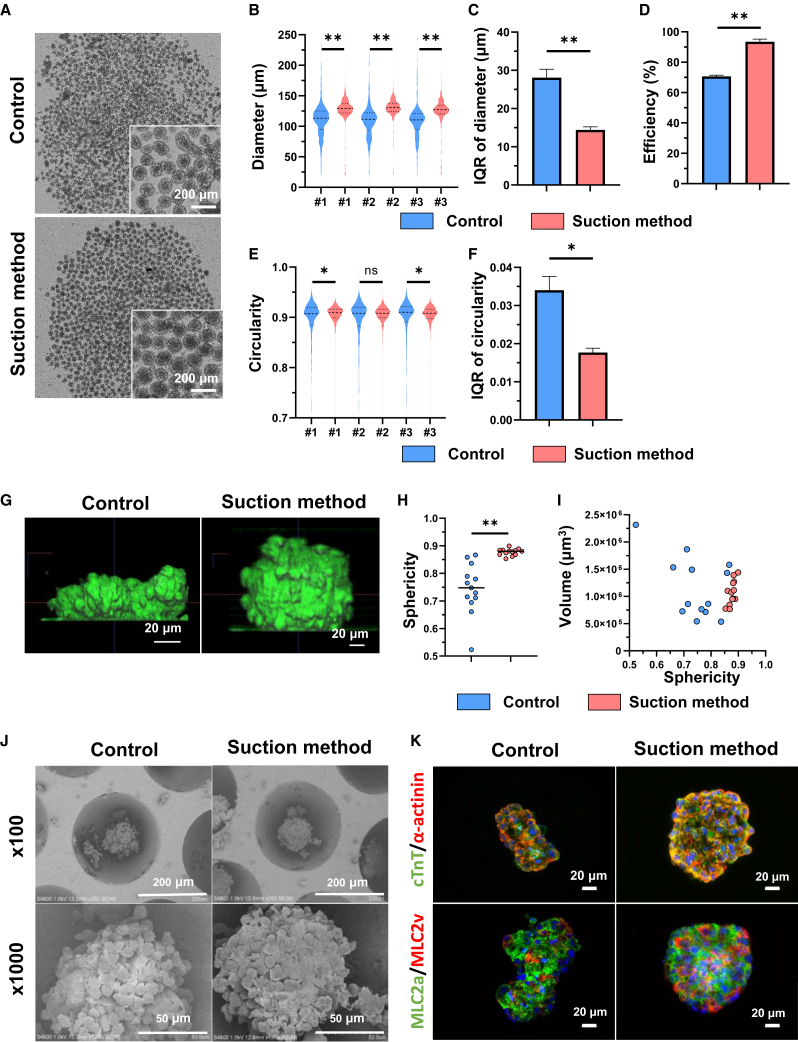
Suction method efficiently produces homogeneous hiPSC-CSs with high sphericity (A) Bright-field microscopy images of hiPSC-derived CSs (hiPSC-CSs) by the control method and the suction method. (B) Violin plot of the diameter of hiPSC-CSs. Brunner-Munzel test, #1: n = 1,065 spheroids, #2: n = 1,036 spheroids, and #3: n = 1,074 spheroids. (C) IQR of the diameter of hiPSC-CSs. Welch’s t test, n = 3. (D) The efficiency of hiPSC-CS production, defined as the percentage of spheroids with a diameter larger than 100 μm. Welch’s t test, n = 3. (E) Violin plot of the circularity of hiPSC-CSs. Brunner-Munzel test, #1: n = 1,065 spheroids, #2: n = 1,036 spheroids, and #3: n = 1,074 spheroids. (F) IQR of the circularity of hiPSC-CSs. Welch’s t test, n = 3. (G) 3D image of hiPSC-CSs observed with 3D imaging using Cell^3^iMager Estier. (H) The sphericity of hiPSC-CSs. Brunner-Munzel test, n = 13 spheroids. (I) The sphericity and volume of hiPSC-CSs were presented on a scatterplot (n = 13 spheroids). The IQR of sphericity for the control method was 0.110, whereas for the suction method, it was 0.00460. Similarly, the IQRs of volume were 8.38 × 10^5^ μm^3^ for the control method and 3.59 × 10^5^ μm^3^ for the suction method. (J) Representative SEM images showing hiPSC-CSs on the substratum. (K) Representative immunofluorescence images for cTnT (green) and α-actinin (red) and for MLC2a (green) and MLC2v (red). Nuclei are stained with Hoechst 33342. The hiPSC data were evaluated using the 253G4 cell line. Data are presented as the mean ± SD. ∗p < 0.05; ∗∗p < 0.01. See also Figures S2 and S4.

We next evaluated the morphological homogeneity of the hiPSC-CSs. We induced hiPSC-CMs from two different hiPSC lines (201B7 and 253G4) and produced hiPSC-CSs 16 and 17 days after the induction of cardiac differentiation. We investigated the homogeneity of hiPSC-CSs on day 2 from the start of culture because the sequential investigation demonstrated that hiPSC-CSs using the suction method were formed stably with little deviation on day 2 (Figures S2A–S2E). The suction method proved successful in producing homogeneous hiPSC-CSs (Figures 2A and S2F**)**. The diameters of hiPSC-CSs were significantly larger when using the suction method than when using the control method (Figures 2B and and S2G). The circularity of hiPSC-CSs tended to be higher in the suction method than in the control method, but certain hiPSC-CSs exhibited significant differences while others did not (Figures 2E and S2J). The deviation in diameter and circularity was significantly reduced within the suction method for both hiPSC-CSs (Figures 2C, 2F, S2H, and S2K). The efficiency of hiPSC-CS production also exhibited a substantial improvement within the suction method when contrasted with the control method, indicating a significant enhancement (Figures 2D and S2I). Sphericity assessment of hiPSC-CSs via 3D imaging indicated a significant increase when utilizing the suction method (Figures 2G and 2H). Scatterplots reflecting the sphericity and volume of the hiPSC-CSs unveiled reduced deviation in both aspects when employing the suction method (IQRs of sphericity: 0.110 in the control method and 0.0460 in the suction method, IQRs of volume: 8.38 × 10^5^ μm^3^ in the control method and 3.59 × 10^5^ μm^3^ in the suction method) compared to the control method (Figure 2I). Similar to hiPSC spheroids, these results indicate that hiPSC-CSs produced using the suction method are more homogeneous and more closely spherical. SEM observation of the hiPSC-CSs revealed that the hiPSC-CSs produced using the suction method were more spherical in shape and exhibited a dense cell structure, whereas those from the control method exhibited a distorted shape with sparse cell density (Figure 2J). To evaluate the cellular characteristics of the hiPSC-CSs, we performed immunostaining of hiPSC-CSs with cardiac troponin T (cTnT), α-actinin, myosin light chain (MLC) 2a, and MLC2v, which are specific markers for CMs. The results revealed no obvious differences between the two methods, indicating that the suction did not functionally affect hiPSC-CSs (Figure 2K). Thus, these results suggest that the suction method can efficiently yield homogeneous hiPSC-CSs with high sphericity.

We proceeded to compare the homogeneity of hiPSC-CSs generated through the suction method with those produced using conventional techniques involving microwell plates and bioreactors, both of which are recognized approaches for spheroid generation. Initially, we sought to determine the optimal conditions in terms of stir rate and cell concentration for the stirred bioreactor. A range of stir rates spanning from 40 to 100 rpm and cell concentrations varying from 1.5 to 2.5 × 10^5^ cells/mL were used (Figures S3A–S3E). While only a limited number of hiPSC-CSs were formed at 40 rpm, the generation of homogeneous hiPSC-CSs was feasible at all cell concentrations within the range of 60–100 rpm (Figure S3E). To identify the most suitable stir rates for achieving the highest homogeneity among hiPSC-CSs, we examined the relationship between spheroid diameter variation, median spheroid diameter, and cell concentration (Figures S3F and S3G). With an intended spheroid diameter of approximately 150 μm, we constructed regression lines for each stirring speed and projected the diameter deviation of hiPSC-CSs from the cell concentration corresponding to the median diameter of the spheroid at 150 μm^7^^,^^8^^,^^15^^,^^47^^,^^48^ (Figures S3F and S3G). Based on these analyses, the condition of 80 rpm was estimated to yield the lowest variation in spheroid diameter, with an associated cell concentration of approximately 7.0 × 10^5^ cells/mL (Figure S3H). Subsequently, we assessed whether hiPSC-CSs with a median diameter of approximately 150 μm could be generated at a cell concentration of 7.0 × 10^5^ cells/mL. However, we observed that while the median diameter of hiPSC-CSs increased up to 6.0 × 10^5^ cells/mL, the median diameter at 7.0 × 10^5^ cells/mL was smaller than that at 6.0 × 10^5^ cells/mL (Figure S3I). Furthermore, several smaller hiPSC-CSs were formed at 7.0 × 10^5^ cells/mL, leading to a larger deviation in hiPSC-CS diameter than that at 6.0 × 10^5^ cells/mL (Figure S3J). These findings indicate that there is no concentration that would result in a larger diameter of hiPSC-CSs than that achieved at the concentration of 6.0 × 10^5^ cells/mL with a stirring speed of 80 rpm. As a result, we opted to proceed with further experiments under the conditions of 80 rpm and a cell concentration of 6.0 × 10^5^ cells/mL.

To assess the homogeneity of hiPSC-CSs produced by the suction and other conventional methods using microwells and bioreactors, we successfully established hiPSC-CSs using each method on day 2 (Figure S4A). In comparison, the diameter of hiPSC-CSs was significantly larger in the suction method than in the bioreactor-based method, whereas it was significantly smaller in the suction method than in the microwell plate method (Figure S4B). The deviation in diameter was significantly smaller using the suction method than that using other conventional methods employing microwells and bioreactors (Figure S4C). While hiPSC-CSs produced using the suction method tended to display higher circularity than those generated through microwell plates, these differences were not consistently statistically significant (Figure S4D). Moreover, no significant difference in circularity deviation was observed among the different methods (Figure S4E). The efficiency of hiPSC-CS production within the suction method was significantly higher than in the bioreactor-based approach, indicating a substantial improvement in efficiency (Figure S4F). Taken together, these findings suggest that hiPSC-CSs generated through the suction method exhibit greater homogeneity than those produced using other conventional methods.

### Suction method can produce homogeneous hiPSC-CSs by large-scale substratum

**Figure 3:**
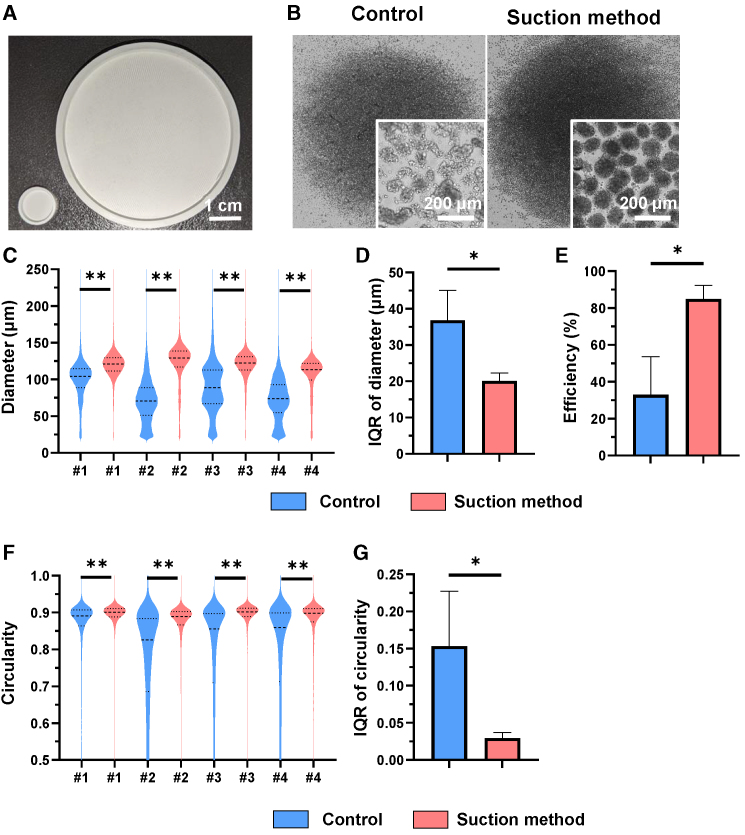
Suction method with large substratum efficiently produces homogeneous hiPSC-CSs with high sphericity (A) Production of large substratum with 39,583 microwells. It is about 9 times larger in diameter than the small substratum on the left used in previous experiments. (B) Production of hiPSC-CSs using large substratum. (C) Violin plot of the diameter of hiPSC-CSs using large substratum. #1–4 indicate the experiment number. Brunner-Munzel test, #1: n = 36,243 spheroids, #2: n = 40,126 spheroids, #3: n = 36,829 spheroids, and #4: n = 28,084 spheroids. (D) IQR of the diameter of iPSC-CSs using large substratum. Welch’s t test, n = 4. (E) The efficiency of hiPSC spheroid production using large substratum, defined as the percentage of spheroids larger than the diameter of 100 μm. Welch’s t test, n = 4. (F) Violin plot of the circularity of hiPSC-CSs using large substratum. #1–4 indicate the experiment number. Brunner-Munzel test, #1: n = 36,243 spheroids, #2: n = 40,126 spheroids, #3: n = 36,829 spheroids, and #4: n = 28,084 spheroids. (G) IQR of the circularity of hiPSC-CSs using large substratum. Welch’s t test, n = 4. The hiPSC data were evaluated using the 253G4 cell line. Data are presented as the mean ± SD. ∗p < 0.05; ∗∗p < 0.01.

Next, we fabricated a large-scale substratum with approximately 40,000 wells to produce a large number of hiPSC-CSs at one time (Figure 3A). We increased the number of hiPSC-CSs that could be obtained at one time from 1,069 to 39,583 hiPSC-CSs by using this substratum (Figure 3B). The diameter and circularity of hiPSC-CSs produced using the suction method were also significantly higher than those of the control method (Figures 3C and 3F). Moreover, the deviation in the diameter was significantly smaller in the suction method (Figure 3D). The efficiency of spheroid production was significantly higher when using the suction method than when using the control method (Figure 3E). The deviation in the circularity was significantly smaller in the suction method than in the control method (Figure 3G). These data are in agreement with those for the small substratum described in Figures 2A–2F and S2F–S2K. Thus, we succeeded in producing approximately 40,000 highly homogeneous hiPSC-CSs with high sphericity at one time.

### Suction method produces functionally homogeneous hiPSC-CSs

**Figure 4:**
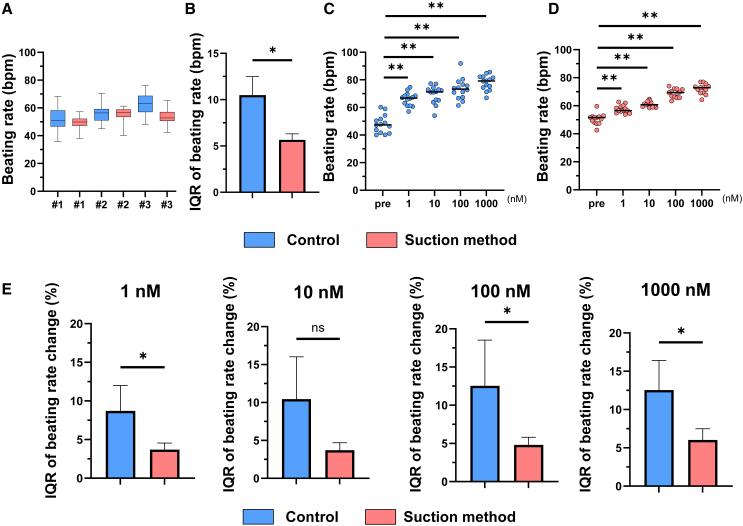
Suction method can produce functionally homogeneous hiPSC-CSs (A) Box-and-whisker diagram of the beating rate of hiPSC-CSs. #1: n = 20 spheroids, #2: n = 20 spheroids, and #3: n = 19 spheroids. We used a small substratum in this experiment. (B) IQR of beating rate of hiPSC-CSs. Welch’s t test, n = 3. (C) Beating rate of hiPSC-CSs at each isoproterenol concentration in the control method. One-way repeated measures ANOVA followed by Dunnett’s multiple comparison test, n = 12. (D) Beating rate of hiPSC-CSs at each isoproterenol concentration in the suction method. One-way repeated measures ANOVA followed by Dunnett’s multiple comparison test, n = 12. (E) IQR of beating rate change of hiPSC-CSs at each concentration of isoproterenol. Welch’s t test, n = 5. The hiPSC data were evaluated using the 253G4 cell line. Data are presented as the mean ± SD. ∗p < 0.05; ∗∗p < 0.01.

We also performed a functional evaluation of hiPSC-CSs produced by the suction method with a small substratum. Autonomous beating of hiPSC-CSs produced by both methods was observed 2 days after starting the culture. Notably, the deviation in the beat rate of hiPSC-CSs was significantly smaller in the hiPSC-CSs produced by the suction method than those produced by the control method (Figures 4A and 4B). When isoproterenol, a nonselective β-agonist, was added in the range of 1 to 1,000 nM, the beating rate increased significantly in both methods, and the deviation of the beating rate change at each concentration was significantly smaller in the suction method for all concentrations except for 100 nM (Figures 4C–4E). These results suggest that the suction method is useful not only for producing hiPSC-CSs for regenerative medicine but also for drug discovery.

### Suction method efficiently produces homogeneous hiPSC-COs

**Figure 5:**
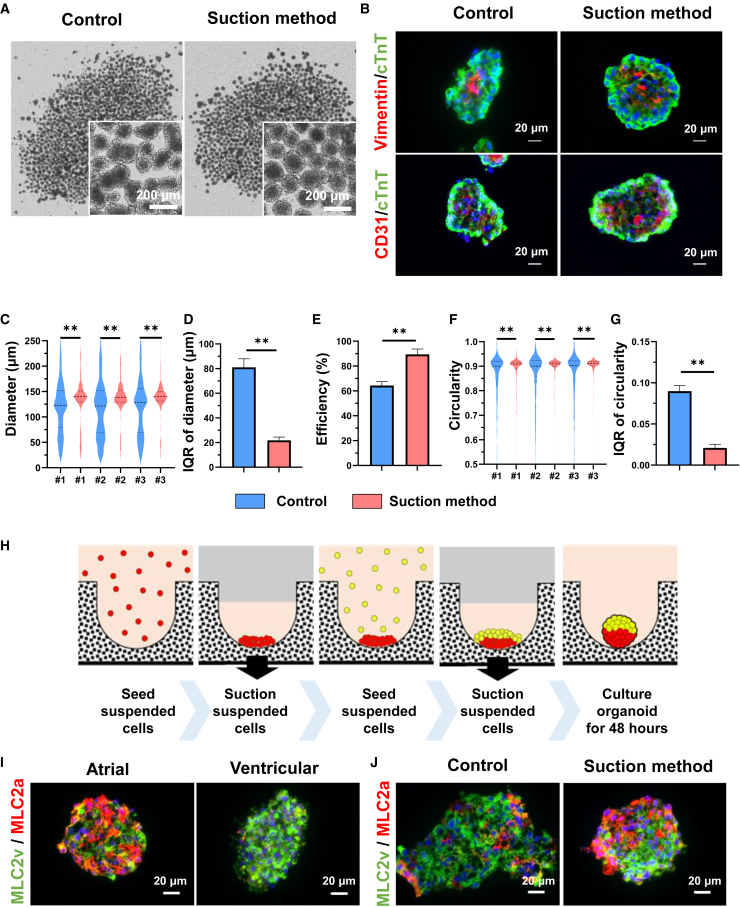
Suction method efficiently produces homogeneous COs and fabricates hiPSC-COs with localized atrial and ventricular tissue (A) Bright-field microscopy images of COs by the control method and the suction method. (B) Representative immunofluorescence images for cTnT (green), vimentin (red), and CD31 (red). Nuclei were stained with Hoechst 33342. (C) Violin plot of the diameter of COs. #1–3 show the experimental number. Brunner-Munzel test, #1: n = 1,078 spheroids, #2: n = 1,014 spheroids, and #3: n = 925 spheroids. (D) IQR of the diameter of COs. Welch’s t test, n = 3. (E) The efficiency of COs production, defined as the percentage of COs larger than the diameter of 100 μm. Welch’s t test, n = 3. (F) Violin plot of the circularity of COs. #1–3 indicate the experiment number. Brunner-Munzel test, #1: n = 1,078 spheroids, #2: n = 1,014 spheroids, and #3: n = 925 spheroids. (G) IQR of the circularity of COs. Welch’s t test, n = 3. (H) Overview of fabrication methods for two-layer organoids. (I) hiPSC-derived cardiac organoids (hiPSC-COs) consist of differentiated atrial and ventricular CMs, respectively. Representative immunofluorescence images for MLC2v (green) and MLC2a (red). Nuclei are stained with Hoechst 33342. (J) Fabrication of hiPSC-COs with localized atrial and ventricular tissue. Representative immunofluorescence images for MLC2v (green) and MLC2a (red). Nuclei are stained with Hoechst 33342. These data relating to hiPSCs were evaluated with 253G4 cell lines. Data are presented as the mean ± SD. ∗∗p < 0.01. See also Figure S5.

We subsequently evaluated the homogeneity of COs produced using the suction method. To generate hiPSC-COs, a mixture of hiPSC-CMs, cardiac fibroblasts (CFs), and human umbilical vein endothelial cells (HUVECs) was employed. Using 2D immunostaining, CFs and HUVECs were confirmed to be vimentin- and von Willebrand factor-positive cells, respectively (Figure S5A). On day 2, hiPSC-COs were successfully formed. The central region of these organoids was marked by the presence of vimentin-positive cells and CD31-positive cells, whereas cTnT-positive cells were primarily identified in the outer part of the hiPSC-COs (Figures 5A and 5B). The hiPSC-COs diameters were significantly larger in the suction method than in the control method (Figure 5C). Notably, the degree of diameter deviation within the hiPSC-COs generated by the suction method was considerably smaller compared to the control method (Figure 5D). Furthermore, the production efficiency of hiPSC-COs was significantly higher in the suction method than in the control method (Figure 5E). Moreover, the circularity of hiPSC-COs produced via the suction method exhibited a noteworthy increase in comparison to the control method. Additionally, the deviation in circularity was significantly reduced within the suction method compared to the control method (Figures 5F and 5G). These results suggest that the hiPSC-COs produced using the suction method exhibited enhanced homogeneity and were more spherical in shape.

### Application of suction method for hiPSC-CO production

We subsequently took advantage of the suction method to engineer hiPSC-COs featuring different types of aggregates strategically positioned at various locations. A method to produce a fused organoid by combining two different types of aggregates in a small well is widely used.^49^^,^^50^ However, it is difficult to produce organoids on a large scale using this method. To produce a large number of hiPSC-COs consisting of two different cell types, we prepared hiPSC-CMs stained red and green using MitoTracker and aspirated the red and green cells successively (Figure 5H). This result showed homogeneous hiPSC-CSs that split into green and red fluorescence (Figure S5B). We confirmed that the production of such specialized hiPSC-CSs was achievable using 96-well plates via centrifugation, whereas they could not be produced without centrifugation (Figure S5C).

Next, we induced atrial and ventricular CMs separately from hiPSCs (Figure S5D). To produce a large number of hiPSC-COs, atrial and ventricular CMs were aspirated successively. We immunostained hiPSC-COs with MLC2a and MLC2v, which are specific markers for atrial and ventricular CMs, respectively (Figure 5I). As a result, hiPSC-COs prepared by the suction method showed clearly separated MLC2a- and MLC2v-positive cell clusters, while hiPSC-COs prepared by the control method showed a mixture of MLC2a- and MLC2v-positive cell clusters (Figure 5J). These results suggest that the suction method can efficiently produce hiPSC-COs using two different cell types.

## Discussion

In this study, we succeeded in producing more efficient and homogeneous hiPSC spheroids and hiPSC-CSs/COs using the suction method than by the conventional method using several cell lines. We also produced hiPSC-CSs with high homogeneity in a large substratum. Furthermore, we succeeded in producing a fused CO model by combining hiPSC-derived atrial and ventricular CMs localized on opposing sides within a single organoid.

To produce homogeneous hiPSC spheroids and hiPSC-CSs/COs, it is important to satisfy the following two requirements: the cells must enter each well evenly, and the cells in the wells must be concentrated together in one place. We succeeded in significantly improving the efficiency of spheroid production and reducing the deviation in the diameter and circularity of spheroids using this suction method (Figures 1D–1I, 2A–2I, and 3A–3G). Our findings indicate that the method of spontaneous sedimentation resulted in notable variation in the number of cells entering each microwell due to the slow sedimentation rate. In contrast, the suction method effectively reduced the variability in cell numbers within microwells by expediting sedimentation through the application of suction to the cell suspension. This distinction was clearly depicted in SEM images, wherein spheroids produced through spontaneous sedimentation were distributed across multiple areas within the wells, whereas those formed via the suction method were more consistently localized (Figures 1M and 2J). We conclude that the suction method sufficiently satisfies the aforementioned requirements, thus facilitating the generation of homogeneous spheroids. In particular, we focused on evaluating the homogeneity of hiPSC-CSs by comparing the suction method with the spontaneous sedimentation method, as well as conventional techniques involving microwell plates and bioreactors. For comparison purposes, we employed microwell plates and conducted centrifugation at 100*g* for 3 min after seeding cell suspensions, mimicking the rapid cell sedimentation achieved by the suction method. Among the methods aside from the suction method, the approach utilizing microwell plates yielded results most comparable to the suction method in terms of homogeneity and production efficiency of hiPSC-CSs (Figures S4A–S4F). However, it is worth noting that the deviation in hiPSC-CS diameter was significantly smaller in the suction method compared to the microwell-plate-based method (Figure S4C). Furthermore, the circularity of hiPSC-CSs tended to be higher using the suction method than using the microwell plate method (Figure S4D). Consequently, these insights suggest that the suction method potentially surpasses conventional methods involving microwell plates, bioreactors, and spontaneous sedimentation, presenting a promising way to produce highly homogeneous spheroids with high production efficiency.

We developed a large-scale substratum with approximately 40,000 microwells to produce a large number of hiPSC-CSs and succeeded in producing approximately 40,000 hiPSC-CSs at one time. The need for generating homogeneous hiPSC-CSs is crucial in the context of clinical applications for cardiac regenerative therapy because it is imperative to avoid the occurrence of internal necrosis within these spheroids. An increased deviation in hiPSC-CS size is linked to a higher proportion of spheroids exceeding the desired size. This has implications since spheroids exceeding 200 μm tend to undergo central necrosis due to hypoxia and nutrient deprivation, which in turn leads to inflammatory responses upon transplantation. Our investigation into the relationship between hiPSC-CS size and the state of internal cells revealed that larger hiPSC-CSs exhibited lower cell density within (Figure S4G). This aligns with previous research that indicates that increased spheroid size results in necrosis due to compromised oxygen and nutrient supply to central cells.^51^^,^^52^^,^^53^ Another practical concern pertains to the potential for hiPSC-CSs with considerable variability to face difficulties during endomyocardial implantation due to clogging within needles. In this study, we successfully achieved the production of homogeneous hiPSC-CSs on a larger-scale substratum. For clinical application of cardiac regenerative therapy, several hundreds of millions of hiPSC-CMs are also required.^3^^,^^12^ Since 40,000 hiPSC-CSs can be produced using the large-scale substratum developed in our study, only about 10 plates will be required for transplantation per person. Furthermore, because there is no technical limitation to increasing the size, it is possible to produce an even larger-scale substratum. Transplantation experiments with spheroids/organoids have been conducted to promote tissue regeneration and angiogenesis not only in the heart but also in the liver, bone, cartilage, spine, and brain, and this technology for mass production of highly homogeneous spheroids/organoids can be useful in a wide range of fields.^16^^,^^17^^,^^18^^,^^19^^,^^20^^,^^21^^,^^22^^,^^23^^,^^24^^,^^25^^,^^26^

Interestingly, the deviation of the beating profile of hiPSC-CSs was significantly smaller in the suction method than that of the control method (Figures 4A and 4B). Beauchamp et al. generated hiSPC-CSs with different cell numbers from hiPSC-CMs and evaluated the differences in beating profiles over time, which demonstrated that the difference in beating rate depended on the size of the hiPSC-CSs.^54^ The hiPSC-CSs produced by the suction method had a smaller deviation in diameter than those produced by the control method, which would be expected to suppress the deviation in the beating rate. In addition, the response of the hiPSC-CSs to isoproterenol showed that the deviation in the beating rate of the hiPSC-CSs was reduced by the suction method (Figure 4E). This may be because the drug penetration was constant owing to the low deviation in size and high sphericity of the hiPSC-CSs produced by the suction method. Therefore, it is suggested that this suction method of producing spheroids may be useful for drug discovery.

Furthermore, as an additional application of the suction method, we successfully generated hiPSC-COs that were composed of separate atrial and ventricular CMs (Figures 5H–5J). The technique of creating fused organoids by combining distinct types of aggregates within a confined space has been widely employed.^49^^,^^50^ However, scaling up this method for producing such spheroids/organoids in larger quantities is challenging. The suction method provides a simple way to generate a substantial quantity of organoids composed of two different cell types. These experiments will contribute to the further advancement of organoid research.

In summary, we have shown that this suction method is highly applicable to a wide range of fields, including hiPSC-based regenerative therapy, drug discovery, and organoid research. We anticipate that this method will lead to yet further research and applications.

### Limitations of the study

The developed suction method can be used to produce spheroids or organoids on a ceramic substratum. However, compared to other conventional methods that utilize microwell plates or bioreactors, it may be difficult to elucidate which factor contributes to spheroid homogeneity, since it is influenced by various factors such as material properties, well size, and microwell number and size. To eliminate this issue, we chose the spontaneous sedimentation method and used the same ceramic substratum with the same number of identical-sized dimples as the control method. We showed that aspiration is the main factor contributing to homogeneity.

In addition, we required a large number of hiPSC-CMs (total density of ∼2.5 × 10^8^ cells) and used different lots in the large-scale experiments. Therefore, there are lot-to-lot variations in the size profiles among experiments. Further studies will be required to reduce lot-to-lot variations in hiPSC-CMs for regenerative therapy and drug discovery.

## STAR★Methods

### Key resources table

**Table:** 

REAGENT or RESOURCE	SOURCE	IDENTIFIER
**Antibodies**		

Anti-TRA-1-60	Merck	Cat#MAB4360; RRID:AB_2119183
Anti-NANOG	abcam	Cat#ab21624; RRID:AB_446437
Anti-OCT4	abcam	Cat#ab200834: RRID:AB_2924374
Anti-SSEA4	SIGMA	Cat#MAB4304; RRID:AB_177629
Anti-α actinin	abcam	Cat#ab50599; RRID:AB_867496
Anti-cardiac Trponin T	abcam	Cat#ab45932; RRID:AB_956386
Anti-MLC2v	abcam	Cat#ab79935; RRID:AB_1952220
Anti-MLC2a	Synaptic Systems	Cat#311-011; RRID:AB_887737
Anti-Vimentin-Cy3	SIGMA	Cat#C9080-2ML; RRID:AB_259142
anti-CD31	abcam	Cat#ab28364; RRID:AB_726362
anti-von Willebrand Factor	abcam	Cat#ab6994; RRID:AB_305689
488 donkey anti-rat IgG	life technologies	Cat#A21208; RRID:AB_2535794
488 goat anti-mouse IgM	life technologies	Cat#A21042; RRID:AB_2535711
488 donkey anti-mouse IgG	invitrogen	Cat#A21202; RRID:AB_141607
647 goat anti-rabbit IgG	life technologies	Cat#A21244; RRID:AB_2535812
647 donkey anti-rabbit IgG	life technologies	Cat#A31573; RRID:AB_2536183

**Chemicals, peptides, and recombinant proteins**		

CHIR99021	Wako	Cat#034-23103
BMP4	R&D systems	Cat#314-BP
IWR-1	SIGMA	Cat#I0161
Retinoic Acid	Sigma	R2625
Y-27632	Wako	Cat#034-24024
Matrigel	Corning	Cat#354230
iMatrix511	Nippi	Cat#892001
iMatrix221	Nippi	Cat#892061
DMEM/F12 GlutaMAX	Thermo Fisher Scientific	Cat#10565-018
AS103C	AJINOMOTO	N/A
AS501	AJINOMOTO	N/A
mTeSR1	STEM CELL Technologies	Cat#ST-85850
RPMI-1640	Wako	Cat#189-02025
MEMα	Thermo Fisher Scientific	Cat#12571–048
D-PBS	Wako	Cat#045-29795
B27-Insulin supplement without Insulin	Thermo Fisher Scientific	Cat#A1895601
FBS	Biowest	Cat#S1560-500
TrypLE Select Enzyme (1X)	Thermo Fisher Scientific	Cat#12563011
2.5g/L-Trypsin/1mmol/I-EDTA Solution	Nacalai Tesque	Cat#35554-64
Sodium Pyruvate Solution	SIGMA	Cat#S8636-100ML
7.5%BSA Fraction V	Thermo Fisher Scientific	Cat#15260-037
Dimethyl sulfoxide (DMSO)	SIGMA	Cat#D2650-100ML

**Critical commercial assays**		

AggreWell 400, 24 well plate starter kit	STEMCELL Technologies	Cat#ST-34450
ABLE 5 mL Disposable Bioreactor	ABLE	Cat#ABBWVS05A
CELLSTAR 96 Well U-bottom plate	Greiner	Cat#650970
Alkaline Phosphatase kit	SIGMA	Cat#86R-1KT
MitoTracker Red FM	Invitrogen	M22425
MitoTracker Green FM	Invitrogen	M7514

**Experimental models: Cell lines**		

253G4 hiPSCs	Provided by CiRA at Kyoto University	N/A
201B7 hiPSCs	Provided by CiRA at Kyoto University	N/A
human neonatal ventricular cardiac fibroblasts (CFs)	Lonza	Cat#CC-2904
human umbilical vein endothelial cells (HUVECs)	Essen BioScience	Cat#4527

**Software and algorithms**		

GraphPad Prism 9	Graphpad	Cat#GPPEAC
BellCurve for Excel	Social Survey Research Information	N/A
Excel	Microsoft	N/A
Cell Motion Imaging System SI8000	SONY	N/A
Cell^3^iMager duos 2	SCREEN	Cat#CC-8300
Cell^3^iMager Estier	SCREEN	Cat#CC-9000

**Other**		

large- or small-scale substratum	CoorsTek	N/A
three-way stopcock	TERUMO	Cat#TS-TR2K
adapter fitting	Nordson MEDICAL	Cat#VFU306
vacuum vessel	AS ONE	Cat#2-7875-01
silicon tube	Masterflex	Cat#96400-16
suction table	CoorsTek	N/A

### Resource availability

#### Lead contact

Further information and requests for resources and reagents should be directed to and will be fulfilled by the lead contact, Shugo Tohyama (shugotohyama@keio.jp).

#### Material availability

This study did not generate new unique reagents.

#### Data and code availability

• All data reported in this paper will be shared by the lead contact upon request.
• This paper does not report original code.
• Any additional information required to reanalyze the data reported in this paper is available from the lead contact upon request.

### Experimental model and study participant details

hiPSCs were maintained in either mTeSR1 (Stemcell Technologies, Vancouver, BC, Canada) or AS103C (Ajinomoto, Tokyo, Japan).^55^^,^^56^ After reaching 70–80% confluency, hiPSCs were passaged as single cells using TrypLE Select Enzyme (1X) (Nacalai Tesque, Kyoto, Japan) and replated in mTeSR1 or AS103C medium with 10 μM Y-27632 (Fujifilm Wako Pure Chemical, Osaka, Japan). After two days, the medium was replaced with medium without Y-27632, and the cells were replaced daily until the next passage. Human neonatal ventricular CFs (Lonza) were maintained with MEMα (Thermo Fisher Scientific) medium including 2% sodium pyruvate (Sigma-Aldrich) and 5% FBS. HUVECs (Essen BioScience) were maintained using EGM2 (Lonza). After reaching 80%–90% confluency, these cells were passaged as single cells using 2.5 g/L Trypsin/1 mmol/L EDTA (Nacalai Tesque), counted using Vi-CELL (Beckman Coulter), subsequently plated in fibronectin-coated dishes.

### Method details

#### Differentiation of hiPSCs to CMs

hiPSCs were differentiated into ventricular CMs as previously described.^9^^,^^10^^,^^11^ Matrigel (Corning, NY, USA) coating (diluted to 1 μL/cm^2^) was incubated for 1–3 h in 4-stack plates or single-stack plates, then plates were seeded with 2-3 × 10^7^ hiPSCs per stack and cultured for 4 days until confluence.

On day 0, the cells were washed with D-PBS (Wako) and incubated in RPMI-1640 (Wako) medium containing 2% insulin-free B27 (Thermo Fisher Scientific, Waltham, MA, USA), 6 μM CHIR99021 (Wako), and 1 ng/mL BMP4 (R&D Systems, Minneapolis, MN, USA). On day 1, the culture medium was changed to RPMI-1640 containing insulin-free B27 and the cells were then incubated for 2 days. On day 3, the culture medium was changed to RPMI-1640 with 2% insulin-free B27 containing 5 μM IWR-1 (Sigma-Aldrich, St Louis, MO, USA) and incubated for 3 days. To induce atrial differentiation, we added 1 μM retinoic acid (Sigma-Aldrich, St Louis, MO, USA) from day 3 to day 10.^57^ On day 6, RPMI-1640 medium was replaced with insulin-free B27, and on day 7, MEMα (Thermo Fisher Scientific) medium with 2% sodium pyruvate (Sigma-Aldrich) and 5% FBS was added for 3 days. On day 10, the cells were detached by adding 2.5 g/L Trypsin/1 mmol/L EDTA (Nacalai Tesque), seeded onto 15 cm dishes coated with Type I Collagen (Iwaki, Shizuoka, Japan) or 1 μL/cm^2^ iMatrix221 (Nippi, Tokyo, Japan) and cultured for 3 days. After that, CMs were metabolically selected for 2–4 days using glucose-free and glutamine-free with lactate media AS501 (Ajinomoto).^11^^,^^13^^,^^58^ After purification, CMs were detached with 2.5 g/L trypsin in 1 mmol/L EDTA and stored at −150°C.

#### Preparation of spheroids

##### Suction and spontaneous sedimentation methods

We assembled the suction device based on the following procedure. First, an adapter fitting (VFU306; Nordson MEDICAL) was attached to the suction port of the suction table for large- or small-scale substratum (provided by CoorsTek Ink.) and a three-way stopcock (TS-TR2K; Terufusion, TERUMO) was connected. Subsequently, the other end of the three-way stopcock was connected to the vacuum vessel (2-7875-01; AS ONE) via a silicon tube (96400-16; Masterflex). The vacuum vessel was evacuated using an aspirator to achieve the desired atmospheric pressure in advance. Before seeding the cells, the substratum provided by CoorsTek Inc. (Golden, CO, USA) was immersed in medium and placed in an incubator. The cells were collected and a suspension of 2.5 × 10^6^ cells/mL and 3.8-5.0 × 10^6^ cells/mL was prepared for hiPSC spheroids and hiPSC-CSs respectively, and 200 μL of each suspension was added to the substratum. In a large substratum experiment, a hiPSC-CM suspension (3.0x10^7^ cells/mL was prepared for hiPSC-CSs, and 10 mL of each suspension was added to the substratum. In the suction method, the substratum was placed on a suction table, and 200 μL or 10 mL of the suspension was transferred to the substratum. The medium in the substratum was aspirated by switching the port on the three-way stopcock. After aspiration of the medium, the port was returned to the three-way stopcock, and the substratum was transferred to 6-well plates or to a 15-cm dish using tweezers. Media was added to these plates/dishes and they were subsequently incubated. In the spontaneous sedimentation method, the substratum was placed directly into the culture plate and 200 μL or 10 mL of the suspension was transferred to the substratum and incubated for 2 h. The culture medium was then added, and incubation was started. The culture period was 2 days, unless otherwise specified.

#### Microwell plates

The initial steps for generating hiPSC-CSs adhered to the manufacturer’s guidelines outlined in the AggreWell 400 (STEMCELL Technologies) plate manual, version 03. To initiate the process, the microwells were treated with 500 μL of an anti-adherence rinse solution (STEMCELL Technologies) followed by centrifugation at 1,300*g* for 5 min, aimed at eliminating any small air bubbles present within the microwells. Subsequently, the anti-adherence rinse solution was aspirated, and each well was washed with 2 mL of MEMα (Thermo Fisher Scientific) medium supplemented with 2% sodium pyruvate (Sigma-Aldrich) and 5% FBS to thoroughly remove any residual anti-adherence rinse. After eliminating 2 mL of the medium, 1 mL of medium was introduced, and hiPSC-CMs were introduced at concentrations of 9x10^5^ cells/mL to facilitate the creation of hiPSC-CSs containing 750 cells each. The AggreWell 400 plate underwent centrifugation at 100*g* for 3 min to confine cells within the microwells, following which the plate was incubated for 2 days.

For the preparation of hiPSC-CSs comprised of either 1,000 or 10,000 cells, the subsequent approach was employed: A total of 1,000 or 10,000 CMs were seeded onto 96-well plates (Greiner) and centrifuged at 300*g* for 3 min. Following this, the cells were incubated, with regular medium replacement every 3 days. Upon completion of a week-long incubation period, the hiPSC-CSs were harvested, and frozen sections were prepared for subsequent analysis of morphology using hematoxylin-eosin staining.

#### Bioreactor

hiPSC-CSs were prepared for culture in 5 mL spinner flasks (Able Biott, Japan). Suspension cultures were established by seeding 1.5 × 10^5^ cells/mL, 2.0 × 10^5^ cells/mL, 2.5 × 10^5^ cells/mL. The spinner flask was placed on a magnetic stirring base plate at the speed of 40, 60, 80 and 100 rpm inside the incubator for 2 days.

#### Preparation of hiPSC-COs

hiPSC-COs were meticulously crafted through the amalgamation of hiPSC-CMs, CFs, and HUVECs in a cell suspension, employing a physiological cell ratio of 95:2.5:2.5. This amalgamated cell population was subsequently gathered, and a suspension of 4.0 × 10^6^ cells/mL for each cell type was prepared. Subsequently, 200 μL of each cell suspension was added to the designated substratum. Thereafter, COs were generated by the suction or spontaneous sedimentation methods, as described in the abovementioned protocol, and incubated for 2 days.

#### Flow cytometry analysis

hiPSC-CMs were dissociated using 0.25% trypsin-EDTA and fixed with 4% paraformaldehyde (MUTO, hereafter abbreviated as PFA) for 20 min. The cells were centrifuged again at 300*g* for 3 min and the supernatant was removed. Triton (0.1%) was added, and the cells were shaken for 30 min, after which 10 mL D-PBS was added. The cells were centrifuged at 300*g* for 3 min, and the supernatant was removed. Then, 1 mL of ImmunoBlock (KAC, Kyoto, Japan) was added, cells were shaken for 30 min, divided into two 1.5 mL tubes (500 μL each), centrifuged at 300*g* for 3 min, and the supernatant was removed. Then, 100 μL each of cardiac troponin T (cTnT) antibody, anti-hu/mou/rat, FITC (Miltenyi Biotec, Cologne, Germany), and REA Control Antibody, human IgG1, FITC (Miltenyi Biotec) were diluted 50-fold in ImmunoBlock. The supernatant was removed after centrifugation at 300*g* for 3 min by adding 1 mL ImmunoBlock. Finally, 300 μL of D-PBS was added and the filtered samples were analyzed using a Gallios 2L6C (Beckman Coulter) flow cytometer.

#### Image analysis of spheroids

The spheroids on the substratum were transferred to a 6-well plate using a P1,000 micropipette and photographed using Cell^3^ imager duos (SCREEN Holdings Co., Ltd.). Spheroids in the images were recognized using a model trained using deep learning. The diameter and circularity of the spheroids were calculated from the area and perimeter of the recognized spheroid regions. The following equations were used to calculate the diameter and circularity of spheroids:

$$Diameter=2\times \sqrt{\frac{Area}{\pi }}$$

$$Circularity=4\pi \times \frac{Area}{{Perimeter}^{2}}$$

In this experiment, all cell aggregates larger than 20 μm were recognized. The Efficiency of spheroid production was defined as the percentage of spheroids larger than 100 μm.

#### Observation with a scanning electron microscope (SEM)

The substratum was washed three times with D-PBS and immersed in 4% PFA for 1 day at 4°C. The cells were then washed three times with D-PBS, immersed in a 2.5% glutaraldehyde/D-PBS solution, and stored overnight at 4°C for pre-fixation. After washing with D-PBS, the cells were post-fixed with a 1% osmium oxide (Wako)/D-PBS solution and left at room temperature for 1 h. After osmium oxide treatment, the cells were washed with pure water and dehydrated by replacing the ethanol solution with increasing concentrations of ethanol (Kanto Chemical, Tokyo, Japan). The samples were then replaced with *t*-butyl alcohol (Wako Pure Chemical Industries) and lyophilized at −50°C. The samples were observed using SEM.

#### Immunohistochemical staining

The spheroids were collected and washed with D-PBS, 4% PFA (MUTO) was added, and the spheroids were incubated at 4°C for one day. The spheroids were washed again with D-PBS and replaced with sucrose (Fujifilm) and D-PBS solution with increasing 10%–30% sucrose concentration in a stepwise manner. The samples were then embedded in O.C.T. compound (Sakura, Tokyo, Japan), quenched with liquid nitrogen, and stored in a freezer at −80°C.

The samples were cut to a thickness of 7 μm using a cryostat (Leica Biosystems, Wetzlar, Germany) and placed on glass slides (Matsunami, Osaka, Japan). The antigen was inactivated with 10 mM citrate buffer (Sigma-Aldrich) adjusted to pH 6.0. The cells were washed three times with D-PBS for 5 min each and incubated in blocking buffer for 45 min. Blocking buffer was prepared by adding 5% Normal Goat Serum (Vector Laboratories, Newark, Ca, USA), 7.5% BSA Fraction V (Gibco, Thermo Fisher Scientific), and 0.1% Triton X-100 (Sigma-Aldrich) in D-PBS. The primary antibodies were mixed with blocking buffer and incubated overnight at 4°C. Subsequently, D-PBS washes were performed three times for 10 min each, and secondary antibodies were added to the blocking buffer and the mix was allowed to stand at room temperature for 1 h. The cells were then washed with D-PBS for 10 min, Hoechst 33342 (Thermo Fisher Scientific) dissolved in blocking buffer was added, and cells were allowed to stand for 10 min at room temperature. After washing with D-PBS again for 10 min, a few drops of mounting medium Fluoromount/Plus (Diagnostic BioSystems, Pleasanton, CA, USA) were added, and the samples were shielded with cover glass (Matsunami). The prepared samples were observed under a fluorescence microscope BZ-X710 (Keyence, Osaka, Japan). The mouse monoclonal antibody against human TRA-1-60 and the mouse monoclonal antibody against human SSEA4 were purchased from Merck KgaA (Darmstadt, Germany). The rabbit polyclonal antibody against human NANOG, the rabbit monoclonal antibody against human OCT4, the rabbit polyclonal antibody against human MLC2v, the rat monoclonal antibody against human α-actinin, the rabbit polyclonal antibody against human cTnT, rabbit polyclonal antibody against human CD31 and rabbit polyclonal antibody against human von Willebrand Factor (vWF) were purchased from Abcam (Cambridge, UK). The mouse monoclonal antibody against human MLC2a was purchased from Synaptic Systems (Göttingen, Germany). The mouse monoclonal antibody against human Vimentin were purchased from Sigma. Alexa Fluor 488-labeled anti-mouse IgM, Alexa Fluor 488-labeled anti-rat IgG, and Alexa Fluor 647-labeled anti-rabbit IgG were purchased from Life Technologies (Carlsbad, CA, USA). Alexa Fluor 488 anti-labelled anti-mouse IgG antibodies were purchased from Invitrogen (Waltham, MA, USA).

#### Alkaline phosphatase (ALP) staining

Undifferentiated hiPSC spheroids cultured on the substratum for 2 days were washed three times with D-PBS together with the substratum. They were then immersed in a 4% PFA solution and allowed to stand overnight at 4°C. To prepare the ALP staining solution, 200 μL each of the sodium nitrite solution from the Alkaline Phosphatase kit (Sigma-Aldrich) and FRV-Alkaline were mixed and allowed to stand for 2 min. Next, 9 mL of Milli-Q water was mixed with 200 μL of Naphthol AS-BI Alkaline. Subsequently, the samples fixed with PFA were washed with Milli-Q water, and then 2 mL of sodium nitrite solution was added to each sample. The cells were kept overnight at room temperature, then observed under a stereomicroscope (LEICA).

#### Cell staining with MitoTracker and multi-coloured hiPSC-CSs

MitoTracker Red FM (Invitrogen) and MitoTracker Green FM (Invitrogen) were used as the reagents. Cells were collected by centrifugation, pellets were suspended in 2 mL of medium containing 500 nM MitoTracker, incubated at room temperature for 30 min, diluted with 10 mL of medium, and centrifuged at 300*g* for 3 min. The supernatant was removed, and the cells were seeded onto culture dishes or a substratum. The method for producing multi-colored hiPSC-CSs through the suction method is detailed in the Results. Multi-colored hiPSC-CSs were created within 96 well plates (Greiner) following this procedure. In the case of the spontaneous sedimentation method, red-stained CMs were initially seeded, succeeded by the introduction of green-stained CMs after a 3 min interval. The resultant mixture was incubated for 2 days. As for the centrifugation method, red-stained CMs were seeded and subsequently subjected to centrifugation at 300*g* for 3 min. Subsequently, green-stained CMs were introduced and similarly subjected to centrifugation at 300*g* for 3 min. The amalgamated cell populations were then incubated for 2 days.

#### Contractile analysis of hiPSC-CSs

The collected hiPSC-CSs were incubated on a 12-well plate coated with fibronectin for approximately 48 h after seeding, and their beating profiles were analyzed using the SI8000 Cell Motion Imaging System (Sony Biotechnology, San Jose, CA, USA).

### Quantification and statistical analysis

All statistical analyses were conducted using GraphPad Prism (GraphPad) and BellCurve for Excel (Social Survey Research Information Co., Ltd.). Statistical significance was determined through the utilization of Welch’s t-test or the Brunner-Munzel test for comparisons between two groups. Welch’s t test was applied to assess the homogeneity and efficiency of spheroids/organoids production. The Brunner–Munzel test was employed to evaluate parameters such as diameter, circularity, and sphericity of the spheroids/organoids. For comparisons involving more than three groups, the one-way repeated measures ANOVA followed by Dunnett’s multiple comparison test, the Brown–Forsythe and welch ANOVA test followed by Dunnett’s T3 multiple tests, or the Kruskal–Wallis test followed by the Dunn’s multiple comparison test were performed. One-way repeated measures ANOVA followed by Dunnett’s multiple comparison test was utilized for assessments related to beating rates in drug response experiments involving hiPSC-CSs. The Brown–Forsythe and Welch ANOVA test, followed by Dunnett’s T3 multiple test, was employed to evaluate the homogeneity of spheroids and the efficiency of spheroid production. Kruskal–Wallis test, followed by the Dunn’s multiple comparison test, was employed for assessing parameters such as diameter and circularity of spheroids. Statistical significance was set at p < 0.05.

## Supplemental information

Document S1. Figures S1–S5

Document S2. Article plus supplemental information
