# Supplementary material for: Scalable production of homogeneous cardiac organoids derived from human pluripotent stem cells
Source: Cell Rep Methods. 2023 Dec 18;3(12):100666. doi: 10.1016/j.crmeth.2023.100666 (PMC10753388; doi:10.1016/j.crmeth.2023.100666)
Supplement: Document S1. Figures S1–S5 [file mmc1.pdf]

Cell Reports Methods, Volume *3*

Supplemental information

Scalable production of homogeneous cardiac organoids derived from human pluripotent stem cells

Taijun Moriwaki, Hidenori Tani, Kotaro Haga, Yuika Morita-Umei, Yusuke Soma, Tomohiko C. Umei, Otoya Sekine, Kaworu Takatsuna, Yoshikazu Kishino, Hideaki Kanazawa, Jun Fujita, Keiichi Fukuda, Shugo Tohyama, and Masaki Ieda

Figure S1

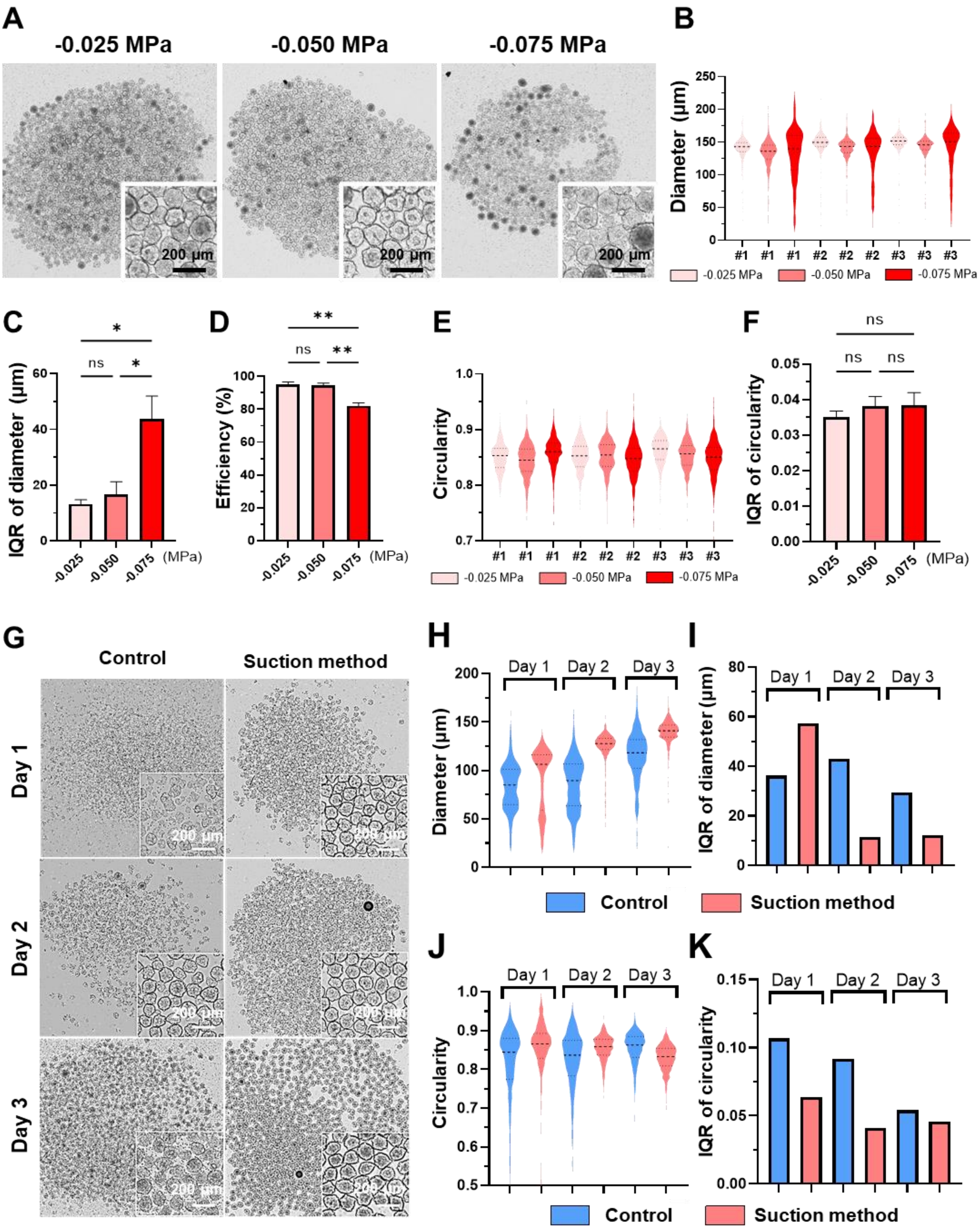
A
-0.025 MPa
-0.050 MPa
-0.075 MPa
200 μm
B
Diameter (μm)
#1 #1 #1 #2 #2 #2 #3 #3 #3
-0.025 MPa
-0.050 MPa
-0.075 MPa
C
IQR of diameter (μm)
ns
*
(MPa)
-0.025
-0.050
-0.075
D
Efficiency (%)
**
ns
E
Circularity
F
IQR of circularity
G
Control
Suction method
Day 1
Day 2
Day 3
H
Diameter (μm)
I
IQR of diameter (μm)
Control
Suction method
J
Circularity
K
IQR of circularity

Fig. S1 Validation of the suction method and collection time of hiPSC spheroids, related to Figure 1.

(A) Brightfield images of day 2 hiPSC spheroids prepared by varying the suction force. (B) Violin plot of the diameter of hiPSC spheroids produced by each suction force. #1-3 indicates the experiment number. (C) Interquartile range (IQR) of the diameter of hiPSC spheroids. Brown–Forsythe and welch ANOVA test followed by Dunnett's T3 multiple test, n = 3. (D) Efficiency of hiPSC spheroids production, defined as the percentage of spheroids with a diameter larger than 100 μm. Brown–Forsythe and welch ANOVA test followed by Dunnett's T3 multiple test, n = 3. (E) Violin plot of the circularity of hiPSC-CSs. #1-3 indicates the experiment number. (F) IQR of the circularity of hiPSC-CSs. Brown-Forsythe and welch ANOVA test followed by Dunnett's T3 multiple test, n = 3. (G) Brightfield microscopy images of hiPSC spheroids each day. These were collected on Day 1, Day 2 and Day 3. (H) Violin plot of the diameter of hiPSC spheroids each day. (I) Interquartile range (IQR) of the diameter of hiPSC spheroids each day. (J) Violin plot of the circularity of hiPSC spheroids each day. (K) IQR of the circularity of hiPSC spheroids at each day. The hiPSC data were evaluated using the 253G4 cell line. Data are presented as the mean ±SD. *p<0.05; **p<0.01.

Figure S2

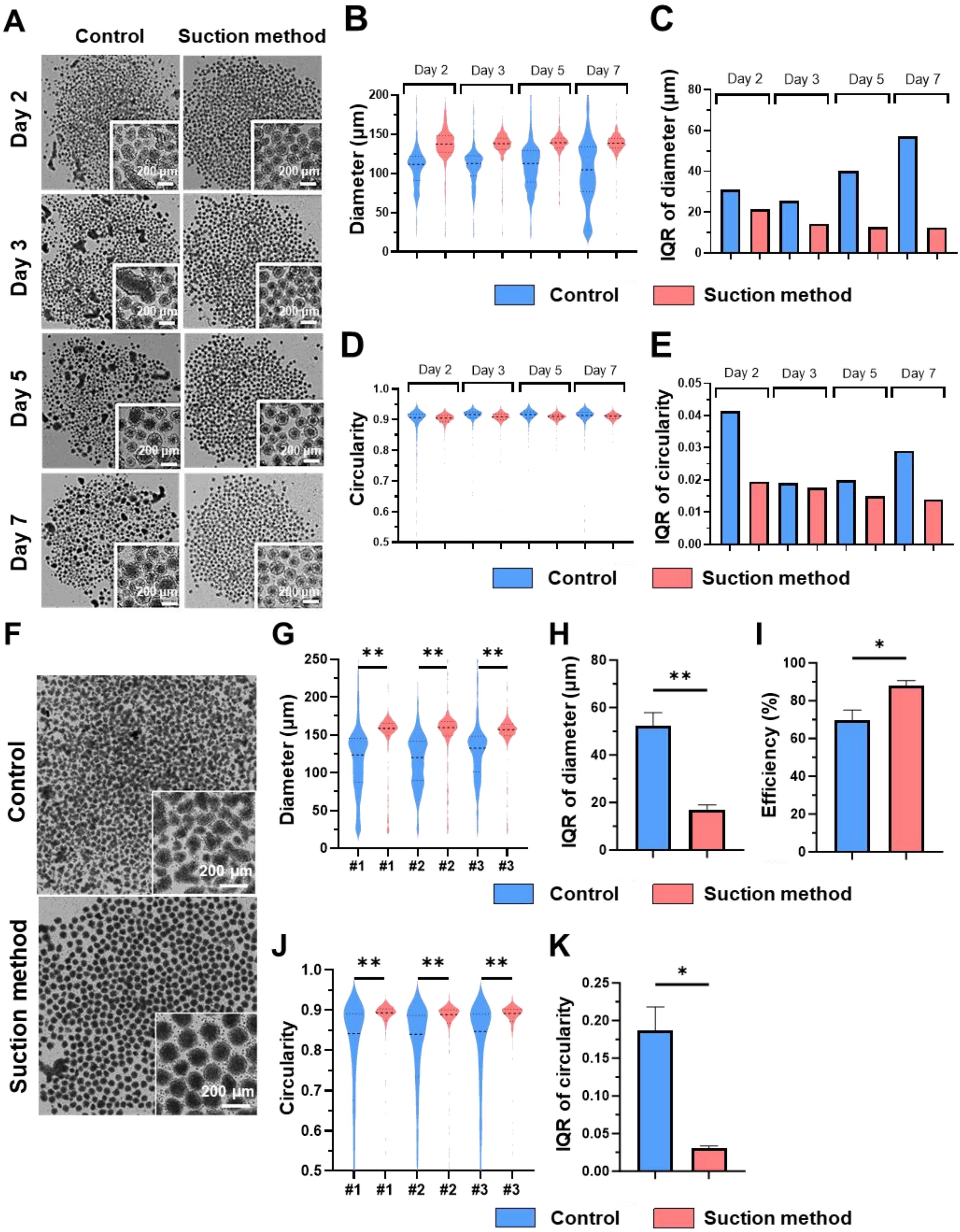

A
Control
Suction method
Day 2
Day 3
Day 5
Day 7
200 μm
B
Diameter (μm)
C
IQR of diameter (μm)
Control
Suction method
D
Circularity
E
IQR of circularity
F
G
#1
#2
#3
H
I
Efficiency (%)
J
K

Fig. S2 Validation of collection time of hiPSC-CSs and producing hiPSC-CSs with another cell line, related to Figure 2.

(A) Brightfield microscopy images of hiPSC-CSs each day. These were collected on Day 2, Day 3, Day 5, and Day 7. (B) Violin plot of the diameter of hiPSC-CSs at each day. (C) Interquartile range (IQR) of hiPSC-CSs diameter each day. (D) Violin plot of the circularity of hiPSC-CSs each day. (E) IQR of hiPSC-CSs circularity each day. (F) Brightfield microscopy images of hiPSC-CSs by the control method and suction method. (G) Violin plot of the diameter of hiPSC-CSs. #1-3 shows experimental number. Brunner–Munzel test, #1: n = 1,004 spheroids, #2: n = 1,046 spheroids, #3: n = 1,056 spheroids. (H) Interquartile range (IQR) of hiPSC-CSs diameter. Welch's t test, n = 3. (I) Efficiency of hiPSC-CSs production, defined as the percentage of spheroids larger than the diameter of 100 μm. Welch's t test, n = 3. (J) Violin plot of the circularity of hiPSC-CSs. #1-3 shows experimental number. Brunner–Munzel test, #1: n = 1,004 spheroids, #2: n = 1,046 spheroids, #3: 1,056 spheroids. (K) IQR of hiPSC-CSs circularity. Welch's t test, n = 3.

(A)–(E), hiPSCs were evaluated using 253G4 cell lines. (F)–(K) hiPSCs were evaluated using 201B7 cell lines. Data are presented as the mean ±SD. *p<0.05; **p<0.01.

Figure S3

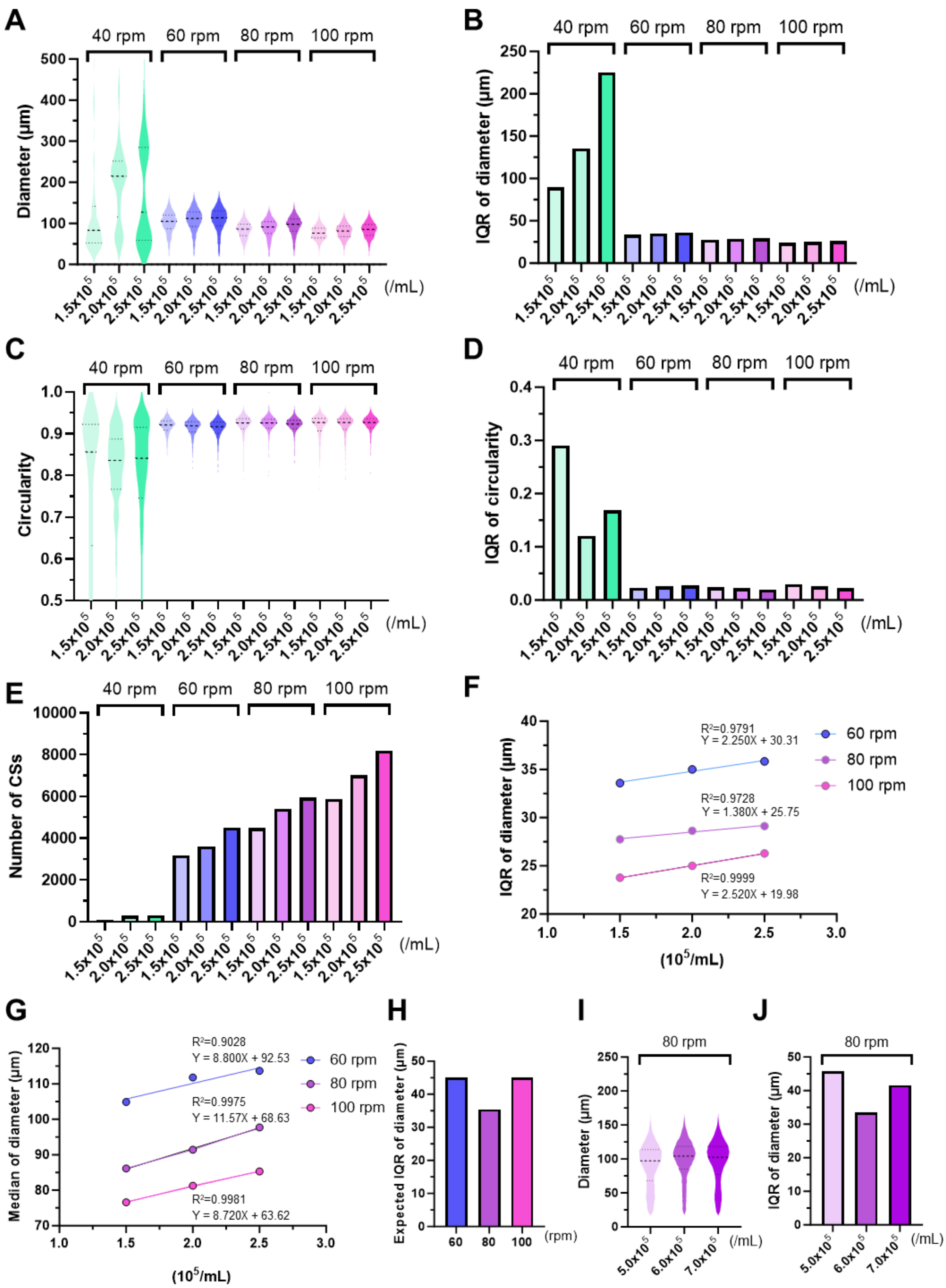

A
40 rpm
60 rpm
80 rpm
100 rpm
Diameter (µm)
(/mL)
B
IQR of diameter (µm)
C
Circularity
D
IQR of circularity
E
Number of CSs
F
R²=0.9791
Y = 2.250X + 30.31
R²=0.9728
Y = 1.380X + 25.75
R²=0.9999
Y = 2.520X + 19.98
60 rpm
80 rpm
100 rpm
IQR of diameter (µm)
(10^5/mL)
G
R²=0.9028
Y = 8.800X + 92.53
R²=0.9975
Y = 11.57X + 68.63
R²=0.9981
Y = 8.720X + 63.62
Median of diameter (µm)
(10^5/mL)
H
Expected IQR of diameter (µm)
(rpm)
I
80 rpm
Diameter (µm)
J
80 rpm
IQR of diameter (µm)

Fig. S3 Cell Concentration and Rotation Speed of Bioreactor, related to Figure 2.

(A) Violin plot of the diameter of hiPSC-CSs produced under varying cell concentrations and rotation speeds. (B) Interquartile range (IQR) analysis illustrating the variation in hiPSC-CS diameter across different cell concentrations and rotation speeds. (C) Violin plot of the circularity for hiPSC-CSs. (D) IQR of circularity variations among hiPSC-CSs. (E) Number of hiPSC-CSs collected. (F)Scatter plots presenting the relationship between median spheroid diameter and cell seeding concentration at different rotation speeds. Straight-line approximations were generated at each of the three data points. (G) Scatter plots indicating the relationship between IQR of spheroid diameter and cell seeding concentration at varying rotation speeds. (H) Expected diameter deviation calculated from Figure S3F, G. (I) Violin plot of the distribution of hiPSC-CS diameter for each cell concentration at 80 rpm.

(J) IQR of hiPSC-CS diameter for each cell concentration at 80 rpm.

The hiPSC data were evaluated using the 253G4 cell line.

Figure S4

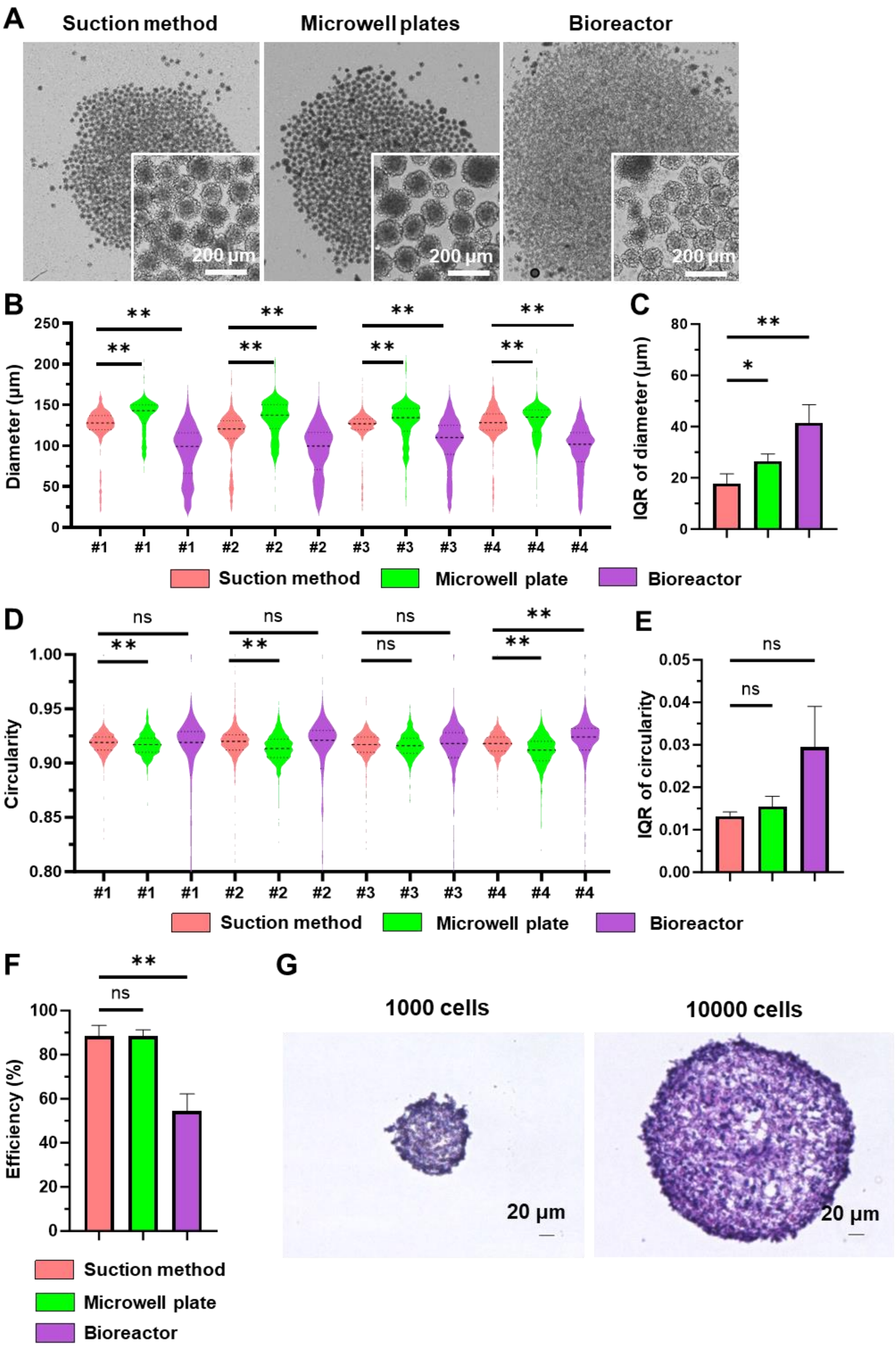
A
Suction method
Microwell plates
Bioreactor
200 μm
200 μm
200 μm
B
Diameter (μm)
250
200
150
100
50
0
**
**
**
**
**
**
**
**
#1 #1 #1 #2 #2 #2 #3 #3 #3 #4 #4 #4
Suction method
Microwell plate
Bioreactor
C
IQR of diameter (μm)
80
60
40
20
0
**
*
D
Circularity
1.00
0.95
0.90
0.85
0.80
ns
**
ns
**
ns
ns
**
**
#1 #1 #1 #2 #2 #2 #3 #3 #3 #4 #4 #4
Suction method
Microwell plate
Bioreactor
E
IQR of circularity
0.05
0.04
0.03
0.02
0.01
0.00
ns
ns
F
Efficiency (%)
100
80
60
40
20
0
**
ns
Suction method
Microwell plate
Bioreactor
G
1000 cells
10000 cells
20 μm
20 μm

Figure S4 Suction method can produce homogeneous hiPSC-CSs as well as or better than microwell plates and bioreactors, related to Figure 2.

(A) Brightfield microscopy images of hiPSC-CSs produced by suction method, microwell-based method and bioreactor-based method. (B) Violin plot of the diameter of hiPSC-CSs. #1-4 indicates the experiment number. Kruskal–Wallis test followed by the Dunn's multiple comparison test, #1: n = 1,139 spheroids, #2: n = 1,150 spheroids, #3: n = 1,175 spheroids, #4: n = 1,119 spheroids. (C) Interquartile range (IQR) of hiPSC-CSs diameter. Brown–Forsythe and welch ANOVA test followed by Dunnett's T3 multiple test, n = 4. (D) Violin plot of the circularity of hiPSC-CSs. #1-4 indicates the experiment number. Kruskal–Wallis test followed by the Dunn's multiple comparison test, #1: n = 1,139 spheroids, #2: n = 1,150 spheroids, #3: n = 1,175 spheroids, #4: n = 1,119 spheroids. (E) IQR of hiPSC-CSs circularity. Brown-Forsythe and welch ANOVA test followed by Dunnett's T3 multiple test, n = 4. (F) Efficiency of hiPSC-CSs production, defined as the percentage of spheroids with a diameter larger than 100 μm. Brown-Forsythe and welch ANOVA test followed by Dunnett's T3 multiple test, n = 4. (G) Brightfield microscopy Images of hematoxylin and eosin staining of hiPSC-CSs composed of 1,000 and 10,000 CMs.

The hiPSC data were evaluated using the 253G4 cell line. Data are presented as the mean ±SD. *$p<0.05$; **$p<0.01$.

Figure S5

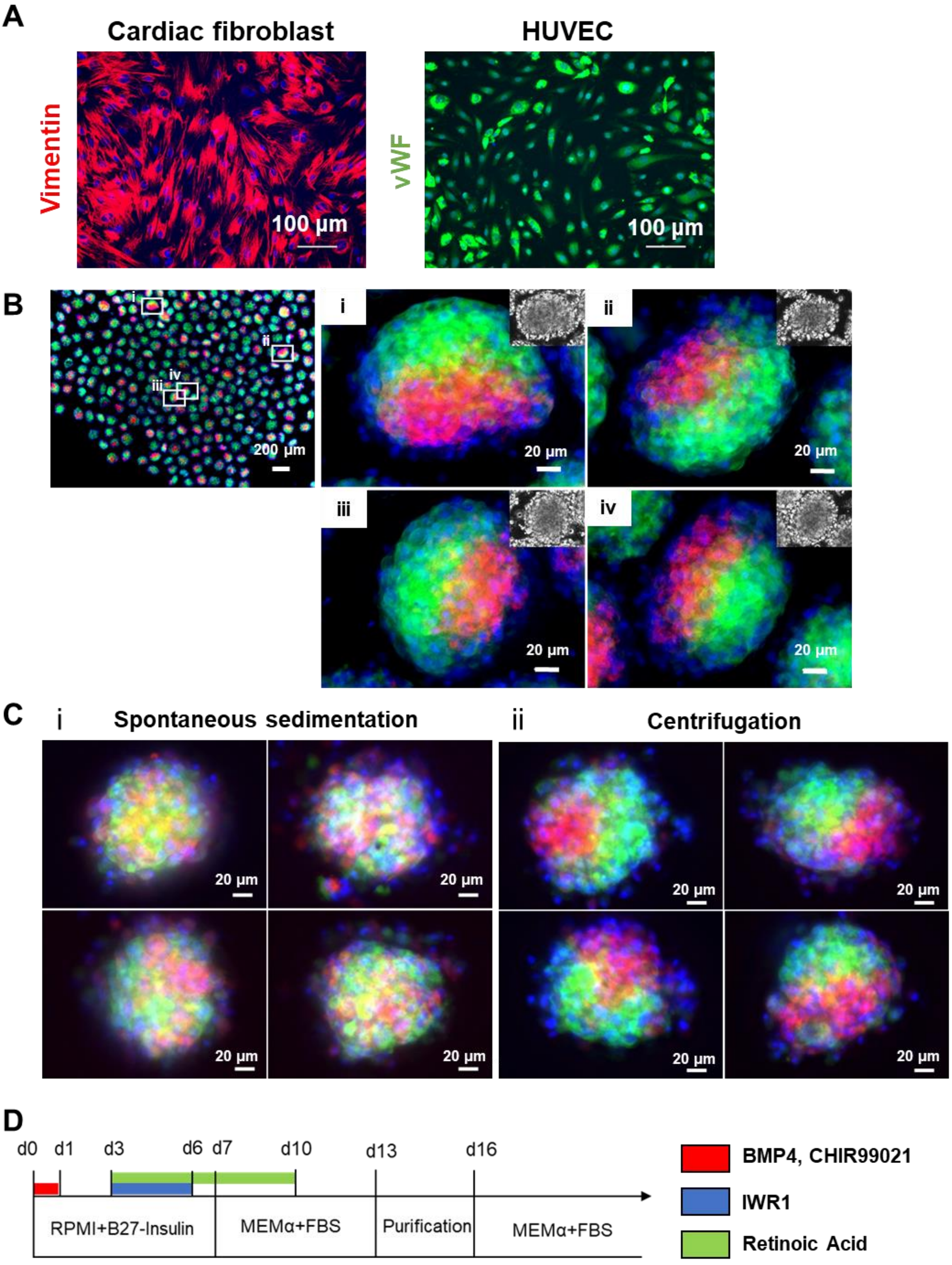
A
Cardiac fibroblast
HUVEC
Vimentin
vWF
100 µm
100 µm
B
i
ii
iii
iv
200 µm
20 µm
20 µm
20 µm
20 µm
C
i
Spontaneous sedimentation
ii
Centrifugation
20 µm
D
d0 d1 d3 d6 d7 d10 d13 d16
RPMI+B27-Insulin
MEMα+FBS
Purification
MEMα+FBS
BMP4, CHIR99021
IWR1
Retinoic Acid

Fig. S5 Application of suction method for production of hiPSC-COs, related to Figure 5.

(A) Representative immunofluorescence images of cardiac fibroblasts (Vimentin) and HUVECs (von Willebrand Factor). Nuclei were stained with Hoechst 33342. (B) Multicolor hiPSC-CSs dyed red and green using MitoTracker. (i) - (iv) are enlarged images of the figure on the left. (C) Fabrication of hiPSC-CSs dyed red and green by using 96 well plate. (i) hiPSC-CSs prepared by seeding red-stained CMs and green-stained CMs with an interval of 3 min. (ii) hiPSC-CSs prepared by seeding red-stained CMs and green-stained CMs with centrifugation at 300g for 3 min. (D) Protocols for differentiation of atrial and ventricular cardiomyocytes (CMs) from hiPSCs.

The hiPSC data were evaluated using the 253G4 cell line.
